# Supplementary material for: Prevalence of male circumcision in four culturally non-circumcising counties in western Kenya after 10 years of program implementation from 2008 to 2019
Source: PLoS One. 2021 Jul 15;16(7):e0254140. doi: 10.1371/journal.pone.0254140 (PMC8281999; doi:10.1371/journal.pone.0254140)
Supplement: S1 File — (PDF) [file pone.0254140.s001.pdf]

Study Title: A Population-Based Survey to Validate Male Circumcision Coverage in Four Counties in Kenya
Principal Investigator: Dr. Anthony Gichangi
IRB No.:
PI Version Date: Version 1; April 15, 2019
JHU IRB No.: 0009667

Appendix 7:STUDY QUESTIONNAIRE

Cluster Number________________________________

Household Number ____

Participant Number ____

Study ID: (String combining CN-HN-client # in the household List)_

Quality Control Checklist

QC1) Interview Status (Circle only one):

Fully Completed - 1

Partially Completed - 2

Refusal or Partially Completed due to Refusal - 3

Total number of visits: _________

QC2) Language of Interview (Circle only one):

Dholuo 1
Kiswahili.. 2
English 3
Other... 97

QC3) Research Assistant Self Check (field), print first name: _______________________

Date: ___________________________

QC4) Field Supervisor Check (field), print surname: ___________________________

Date: ___________________________

QC5) Field Supervisor Sit-in (field), print surname: ___________________________

QC6) Field Supervisor HH Back-check (field), print surname: ___________________________

QC7) Field Manager Check (field), print surname: ___________________________

Date: ___________________________

QC8) Field Manager HH Back-check (field), print surname: ___________________________

QC9) # of missing values found & fixed by Field Supervisor: ____________________

Supervisory Comments: ____

Questionnaire Entry Completed: ___________________________

Date: ___________________________

Section 1: DEMOGRAPHIC AND BACKGROUND QUESTIONS

QNo	Question	Responses	Codes	Go To
101	How old were you at your last birthday (years)?<br>DOB	__________ Years<br>________________		
102	What is your current marital status?	not married, without regular partner (single)	1	
		not married, with regular live-in partner	2	
		not married, regular partner, not living with partner	3	
		married, living with wife	4	
		married, not living with wife	5	
		widow	6	
		divorced/separated	7	
		don't know	97	
		refused to answer	98	
103	What is your ethnic group	EMBU=1<br>KALENJIN=2<br>KAMBA=3<br>KIKUYU=4<br>KISII=5<br>LUHYA=6<br>LUO=7<br>MASAI=8<br>MERU=9<br>MIJIKENDA=10<br>SOMALI=11<br>TAITA TAVETA=12<br>SWAHILI=13<br>OTHERS=96		

QNo	Question	Responses	Codes	Go To
		______________________<br>(SPECIFY)<br><br>…………………………………		
104	How long have you lived here in<br><br>[name of community/ town neighborhood/ village]?<br><br>[WRITE IN OR CIRCLE AS APPLICABLE]	NUMBER OF YEARS ________<br>or<br>Less than 1 year<br>do not know/cannot say	 <br>1<br>97	
105	What religion do you belong to?	Christianity<br>Muslim<br>Judaism<br>Traditional Religion<br>none/agnostic<br>other (specify): ______________	1<br>2<br>3<br>4<br>5	
106	What is the highest level of school you attended?	none<br>lower primary (Grades 1-4)<br>upper primary (Grades 5-7)<br>junior secondary (Grades 8-10)<br>senior secondary (Grades 11-12)<br>greater than secondary	1<br>2<br>3<br>4<br>5<br>6	107<br>106a<br>106a<br>106a<br>106a<br>106a
106a	[If attended school to 106] What is the highest grade/level you completed at that level?	______________		
107	Have you done any work in the last 12 months for which you received cash or a form of non-monetary payment?	yes<br>no<br>don't know<br>refused to answer	1<br>2<br>97<br>98	
108		yes	1	

QNo	Question	Responses	Codes	Go To
	Have you done any work in the last seven days for which you received cash or a form of non-monetary payment?	no<br>don’t know<br>refused to answer	2<br>97<br>98	
109	How would you describe your current employment situation/occupation? [*Circle only one*]	formal employment<br>informal/self-employment<br>actively seeking work<br>unemployed<br>student (full time)<br>Student (part-time)<br>Other, specify_________	1<br>2<br>3<br>4<br>5<br>6<br>#	
110	**Have you done any work in the last 12 months for which you traveled to another county outside the county of your residence?**	yes<br>no<br>don’t know<br>refused to answer	1<br>2<br>97<br>98	
111	**Have you done any work in the last seven days for which you traveled to another county outside the county of your residence?**	yes<br>no<br>don’t know<br>refused to answer	1<br>2<br>97<br>98	
112	**If you are currently looking for employment, in the last 1 month, has your search taken you to another county outside the county of your residence?**	yes<br>no<br>Not looking for employment<br>don’t know<br>refused to answer	1<br>2<br>3<br>97<br>98	Go to 113
113	**[If yes] to which county/counties**	……………………………………………<br>………………..County	#	

Section 2: MALE CIRCUMCISION: KNOWLEGDEOPINIONS

QNo	Question	Responses	Codes	Go To
201	Have you ever heard of 'male circumcision' before I arrived here today?' [RA: if respondent says no to term in language of interview, mention the phrase in English]	yes<br>no [confirm using English term]	1<br>2	202a<br>Sec 3
202	[If yes to 201] What have you heard? [Circle all that apply]	remove the foreskin<br>protects man from HIV<br>much pain during operation<br>much pain after operation<br>loses sensitivity<br>gains sensitivity<br>circumcised penis is cleaner<br>do not know/cannot say	1<br>2<br>3<br>4<br>5<br>6<br>7<br>97	
203	Do you know of any males in your immediate or extended family who have been circumcised?	yes<br>no<br>do not know/cannot say	1<br>2<br>97	204a<br>Sec 3<br>Sec 3
204a	[*If Yes*] How many?	Enter No: ____________		204b
204b	[If yes to 203] If more than one male, consider the relative closest to you. Was he circumcised at a health facility or by other practitioners??	health facility<br>traditional practitioner<br>religious leaders<br>family elders<br>other (specify) __________________<br>do not know/cannot say	1<br>2<br>3<br>4<br>#<br>97	

Section 3: SERVICE DELIVERY EXPERIENCES AND PERCEPTIONS OF CIRCUMCISED MEN

QNo	Question	Responses	Codes	Go To
301	Are you circumcised?	Yes<br>No<br>do not know/cannot say	1<br>2<br>97	302<br>301a<br>Sec. 4
301.a	*[If not circumcised*] Why have you not gone for circumcision?	Tradition/culture<br>Nobody has told me about it<br>My personal decision<br>Doctor advised for health reasons<br>Other (specify)----------	**1**<br>2<br>3<br>4<br>**7**	All: go Sec. 4
302	Why did you decide to go for circumcision?<br>(Select all that apply)	Tradition/culture<br>Recruited by someone<br>My personal decision - reduce risk of HIV<br>My personal decision - reduce risk of STIs<br>My personal decision – improve sexual performance<br>Doctor advised for health reasons<br>Other (specify)----------<br>Do not know/cannot say	1<br>2<br>3<br>4<br>5<br>6<br>#<br>97	
303	What was your age at circumcision?	adult<br>infant<br>young child aged 1-4<br>adolescent aged 5-14<br>teenager aged 15-17<br>do not know/cannot say	1<br>2<br>3<br>4<br>5<br>97	303a<br>304<br>304<br>304<br>303.a<br>304

QNo	Question	Responses	Codes	Go To
303.a	How long ago were you circumcised?	Last I week<br>Last 1 month<br>Last 6 months<br>Last 1 year<br>If more than 1 year ago, write year …<br>**do not know/cannot say**	1<br>2<br>3<br>4<br>5<br>97	
304	Where were you circumcised?	Facility name/Location:___________<br>At home<br>do not know/cannot say	1<br>2<br>97	 307
305	Were you circumcised by a health worker or a traditional healer/non health worker?	health worker<br>traditional healer<br>do not know/cannot say	1<br>2<br>97	306a-c<br>307<br>307
306a	Did the health worker inform you about the risks and benefits of circumcision before you consented for the procedure?	yes<br>no<br>do not know/cannot say	1<br>2<br>97	
306b	Did the health worker give you any instructions around your recovery period related to how long to abstain from sex, whether you needed to go for follow-up check-ups, etc.?	yes<br>no<br>do not know/cannot say	1<br>2<br>97	
306c	What did s/he tell you to do/not to do?<br>[*Select all that apply*]	Abstain from sex<br>Follow-up check up<br>Wound care<br>Some risk for HIV even if circumcised<br>Healing period<br>No instructions given<br>Do not know/ cannot remember<br>Others (Specify): _____________	1<br>2<br>3<br>4<br>5<br>6<br>97<br>#	

QNo	Question	Responses	Codes	Go To
307	Which of the following statements best describes your discussions with other men about male circumcision?<br><br>ONLY ONE ANSWER	I don't talk to other men about male circumcision	1	309
		When other men bring up the subject, I talk to them about male circumcision, but only with my friends or family members	2	308
		When other men bring up the subject, I talk to them about male circumcision, whether they are my friends or family members or even someone I don't know well	3	308<br>308
		I bring up the subject with other men to encourage them to get circumcised, but only with my friends or family members	4	308
		I bring up the subject with other men to encourage them to get circumcised, even if they are someone I don't know well	5	
308	ASK IF PRIOR QUESTION = 2-5<br><br>What do uncircumcised men ask you about your experience getting circumcised?<br><br>READ ALL STATEMENTS<br><br>MULTIPLE ANSWERS POSSIBLE<br><br>ROTATION OF STATEMENTS	About where you got it done	1	
		About the counseling session before getting it done	2	
		About any pain you felt during the procedure	3	
		About the instructions you were given after the procedure	4	
		About any pain you felt during the healing time	5	
		About the impact of the healing process on your daily life, like working or going to school	6	
		About the impact of the healing process on your sex life, if you have a partner	7	
		About the reaction of your partner after seeing your circumcised penis for the first time	8	
		About any embarrassing moments you felt after getting circumcised	9	
		About any moments of pride you felt after getting circumcised	10	

QNo	Question	Responses	Codes	Go To
309	Did you have any problems or complications in the days following circumcision procedure?	yes<br>no<br>do not know/cannot say	1<br>2<br>97	309a<br>310<br>310
309a	[If 'had an adverse event' to 404]<br>What type of problem or complication did you experience?<br>[ALLOW MULTIPLE RESPONSE]	Abnormal pain<br>Excessive swelling<br>Hematoma<br>Bleeding<br>Infection<br>Difficulty urinating<br>Delayed wound healing<br>Problems with appearance<br>Injury to glans<br>Erectile dysfunction<br>other (specify) _______________	1<br>2<br>3<br>4<br>5<br>6<br>7<br>8<br>9<br>10<br>#	
309b	How was the problem or complication mentioned above resolved?	Received treatment at the health facility where MC took place<br>Received treatment at another facility<br>AE resolved without treatment<br>Self-treated AE<br>Received treatment from a pharmacist (did not see clinician)<br>Received treatment from a traditional healer<br>Adverse event has not been resolved<br>Do not know/cannot say	1<br>2<br>3<br>4<br>5<br>6<br>7<br>97	
309c	How long did it take to resolve?	1-3 days<br>4-6 days<br>More than 7 days<br>Adverse event has not been resolved	1<br>2<br>3<br>4	

QNo	Question	Responses	Codes	Go To
		Do not know/cannot say	97	
310	What do you regard as the main benefits to you, if any, since being circumcised? [Circle up to 3 responses]<br><br>[DO NOT READ LIST, SELECT RESPONSES MENTIONED]	none [circle by itself]<br>it is just better overall [circle by itself]<br>protect myself against HIV<br>protect myself against other STIs<br>better hygiene<br>enhanced sexual pleasure<br>consistent with tradition<br>consistent w/religious beliefs/practices<br>do not know/cannot say [circle by itself]	1<br>2<br>3<br>4<br>5<br>6<br>7<br>8<br>97	
311	Would you recommend male circumcision to your friends or family?	yes<br>no<br>do not know/cannot say	1<br>2<br>97	
312	Have you referred anybody for circumcision?	yes<br>no<br>do not know/cannot say	1<br>2<br>97	
313	Why would you not recommend circumcision to a friend/family?	yes<br>no<br>do not know/cannot say	1<br>2<br>97	

Section 4: VERIFICATION OF CIRCUMCISION STATUS

RA: Seek signed consent from eligible participants to verify circumcision status.

401	Circumcision Status Upon Physical Examination	Fully Circumcised<br>Partially Circumcised<br>Uncircumcised	1<br>2<br>3	End<br><br>402
402	**When, if ever, would you be willing to get circumcised? (Select one)**<br>**READ SCALE**<br>**SELECT ONLY ONE ANSWER**	Never<br>Within the next 2 weeks<br>In 2 to 4 weeks<br>In 1 to 3 months<br>In 4 to 6 months<br>In 7 to 12 months<br>After 12 months from now	0<br>1<br>2<br>3<br>4<br>5<br>6	

End of interview. Thank the person for their cooperation. Circle level of co-operation below. If there are any responses that you think are unreliable, write under "comments" which questions and why you think that they are unreliable.

500) Level of co-operation

____ 1. high ______ 2. medium ____ 3. low

Comments on <u>any</u> issues/questions
