# Supplementary material for: Prevalence of male circumcision in four culturally non-circumcising counties in western Kenya after 10 years of program implementation from 2008 to 2019
Source: PLoS One. 2021 Jul 15;16(7):e0254140. doi: 10.1371/journal.pone.0254140 (PMC8281999; doi:10.1371/journal.pone.0254140)
Supplement: S2 File — (PDF) [file pone.0254140.s002.pdf]

Peana hati za idhini kwenye ***Microsoft Word PEKEE***<br><br>*Wacha wazi kwa matumizi ya ofisi ya IRB.*	***Matumizi ya Ofisi ya IRB Pekee:***<br>Tarehe ya Idhinisho:<br>Tafsiri ya idhini ya IRB iliyoidhinishwa :<br>Jina la PI:<br>Nambari ya IRB.

Mada ya Utafiti: Aua ijikitayo kwa watu ili kuhalalisha kutahiriwa kwa wanaumme katika Kaunti nne nchini Kenya.
Mtafiti mkuu: Dr. Anthony Gichangi
Nambari ya IRB.:
Tarehe ya Tafsiri ya PI: Tafsiri 1; April 15, 2019

Fomu ya Idhini ya Wazazi

Hojaji ya Utafiti

Nambari ya nguzo________________________________

Nambari ya Nyumba ____

Orodha ya kuthibiti kiwango cha ubora

QC1) Hadhi ya mahojiano (Chorea moja tu):

Imekamilika yote - 1

Baadhi yamekamilika - 2

Alikataa au baadhi yamekamilika kwa sababu ya kukataa - 3

Nambari ya jumla ya ziara: _________

QC2) Lugha ya mahojiano (Chorea moja tu):

Dholuo 1
Kiswahili 2
Kiingereza 3
Nyingine 97

QC3) Kujichunguza binafsi kwa msaidizi wa utafiti (nyanjani), Andika jina la kwanza:

____

Tarehe: ___________________________

QC4) Angalizo la msimamizi wa nyanjani (nyanjani), Andika jina la familia/la baba:

____

Tarehe: ___________________________

QC5)Maangalizi ya msimamizi mtendaji wa nyanjani, Andika jina la familia/la baba:

____

QC6) Maangalizi ya msimamizi wa nyanjani (nyanjani), Andika jina la familia/la baba:

____

QC7) Maangalizi ya mkurugenzi wa nyanjani (nyanjani), Andika jina la familia/la baba:

____

Tarehe: ___________________________

QC8) Maangalizi ya Mkurugenzi wa nyanjani (nyanjani), Andika jina la familia/la baba:

____

QC9) # vitu vilivyokosekana na kurekebishwa na msimamizi: _____________________

Maoni ya usimamizi: ____

Nambari ya kumalizika kwa hojaji: ___________________________

Tarehe: ___________________________

*Peana hati za idhini kwenye **Microsoft Word PEKEE***<br><br>*Wacha wazi kwa matumizi ya ofisi ya IRB.*	***Matumizi ya Ofisi ya IRB Pekee:***<br>Tarehe ya Idhinisho:<br>Tafsiri ya idhini ya IRB iliyoidhinishwa :<br>Jina la PI:<br>Nambari ya IRB.

Sehemu 1: MASWALI YA DEMOGRAFIA NA USULI

Namba ya Swali	Swali	Majibu	Kodi	Nenda
101	Ulikuwa na umri upi kwenye siku yako ya kuzaliwa kwako ya mwisho? (miaka)<br>Tarehe ya kuzaliwa	__________ Miaka<br>_________________		
102	Hadhi yako ya ndoa kwa sasa hivi ni gani?	Sijaolewa, bila mwenza thabiti (hayuko kwenye mahusiano)<br>Hajaolewa, na mwenza thabiti wanayeishi naye<br>Hajaolewa, mwenza thabiti, haishi na mwenza<br>Kwenye ndoa, anaishi na mke<br>Kwenye ndoa, haishi na mke<br>Mjane<br>Wametalikiana/wametengana<br>Sijui<br>Alikataa kujibu	1<br>2<br>3<br>4<br>5<br>6<br>7<br>8<br>9<br>97<br>98	
103	Wewe ni kabila gani?	EMBU=1<br>KALENJIN=2<br>KAMBA=3<br>KIKUYU=4<br>KISII=5<br>LUHYA=6<br>LUO=7<br>MASAI=8<br>MERU=9<br>MIJIKENDA=10		

		SOMALI=11<br>TAITA TAVETA=12<br>SWAHILI=13<br>LINGINE=96<br>______________________<br>(TAJA HUSIKA)<br>………………………………….		
104	Umeishi huku kwa muda upi?<br>[jina la jamii/mji ulioko karibu/kijiji?]<br>[ANDIKA AU CHOREA YANAYOHUSIKA]	NAMBARI YA MIAKA________ au<br>chini ya mwaka mmoja/hajui<br>hawezi kusema	1<br>97	
105	Wewe ni wa dini gani ?	Mkristo<br>Muislamu<br>Myuda<br>hakuna/agnostic<br>Nyingine (taja husika): ______________	1<br>2<br>3<br>4<br>5	
106	Umehitimu kiwango kipi cha juu zaidi cha masomo?	Hakuna<br>Miaka ya kwanza ya shule ya msingi (Gredi 1-4)<br>Miaka ya mwisho ya shule ya msingi (Gradi 5-7)<br>Miaka ya kwanza ya shule ya upili (Gradi 8-10)<br>Miaka ya mwisho ya shule ya upili (Gradi 11-12)<br>Zaidi ya shule ya upili	1<br>2<br>3<br>4<br>5<br>6	107<br>106a<br>106a<br>65a<br>106a<br>106a

106a	[Iwapo alihitimu masomo mpaka kiwango hicho 106] ni kiwango kipi cha juu zaidi masomo alichohitimi ?	_______________		
107	Umewahi kufanya kazi na ukalipwa kwa njia ya pesa au malipo mengine ambayo sio pesa ndani ya miezi 12 iliyopita	ndio<br>la<br>sijui<br>alikataa kujibu	1<br>2<br>97<br>98	
108	Umewahi kufanya kazi na ukalipwa kwa njia ya pesa au malipo mengine ambayo sio pesa ndani ya siku saba zilizopita?	ndio<br>la<br>sijui<br>alikataa kujibu	1<br>2<br>97<br>98	
109	Unaweza kueleza vipi aina ya ajira yako ya sasa?<br>[*Chorea moja tu*]	Kwenye ajira<br>Kujiajiri<br>anatafuta ajira<br>Hana ajira<br>Mwanafunzi (wakati wote)<br>Mwanafunzi (anasoma katika kipindi fulani)<br>Nyingine,taja husika _________	1<br>2<br>3<br>4<br>5<br>6<br>#	
110	**Umefanya kazi yoyote ndani ya miezi 12 iliyopita ambamo ulisafiri kwenda Kaunti nyingine ambayo sio kaunti yako ya kuishi?**	ndio<br>la<br>sijui<br>alikataa kujibu	1<br>2<br>97<br>98	
111	**Umefanya kazi yoyote ndani ya siku 7 zilizopita ambamo ulisafiri kwenda kaunti nyingine ambayo sio kaunti unayoishi?**	Ndio<br>la<br>sijui<br>alikataa kujibu	1<br>2<br>97<br>98	
112	**Iwapo unatafuta kazi kwa sasa, ndani ya mwezi 1 uliopita, kutafuta kwako kazi kumekupeleka nje ya kaunti**	ndio<br>la	1<br>2	113

	**unayooishi?**	Sitafuti kazi<br>sijui<br>alikataa	3<br>97<br>98	
113	**[Iwapo ndio] kwenda kaunti gani?**	……………………………………………<br>………………..Kaunti	#	

Peana hati za idhini kwenye ***Microsoft Word PEKEE***<br><br>*Wacha wazi kwa matumizi ya ofisi ya IRB.*	***Matumizi ya Ofisi ya IRB Pekee:***<br>Tarehe ya Idhinisho:<br>Tafsiri ya idhini ya IRB iliyoidhinishwa :<br>Jina la PI:<br>Nambari ya IRB.

Sehemu 2: KUTAIRI WANAUMME: KUFAHAMU MAONI

Namba ya swali	Maswali	Majibu	Kodi	Nenda
201	Umewahi kusikia kuhusu "kutahiri wanaumme" kabla nije hapa leo hii? [RA: iwapo mshiriki atasema hapana kwa neno hilo kwenye lugha ya mahojiano, rudia neno hilo kwa kiingereza]	Ndio<br>La [Thibitisha kwa kutumia neno la kiingereza]	1<br>2	202a<br>Seh 3
202	[Iwapo ndio kwa 201] Umeskia nini [Chorea husika]	Kutoa ngozi ya juu ya uumme<br>Unamzuia mwanaumme kwenye maambukizi ya HIV<br>Uchungu mwingi wakati wa kufanyika<br>Uchungu mwingi baada ya kufanyika<br>Inapoteza unyeti<br>Inaleta unyeti<br>Uumme uliotahiriwa ni safi<br>Sijui/siwezi kusema	1<br>2<br>3<br>4<br>5<br>6<br>7<br>97	
203	Unawafahamu wanaumme wowote katika familia yenu ndogo au kubwa ambao wametahiriwa ?	ndio<br>la<br>Sijui/siwezi kusema	1<br>2<br>97	204a<br>Seh 3<br>Seh 3
204a	[*Iwapo Ndio*] Wangapi?	Andika Nambari: ___________		204b
204b	[Iwapo Ndio kwa 203] Iwapo zaidi ya mwanaumme mmoja, chukulia yule ambaye ana uhusiano wa karibu na wewe. Alitahiriwa katika kituo cha Afya au na wahudumu wengine?	Kituo cha Afya<br>Mhudumu wa Kienyeji<br>Kiongozi wa dini<br>Viongozi wa familia<br>Mwingine(taja husika)	1<br>2<br>3<br>4<br>97	

*Peana hati za idhini kwenye **Microsoft Word PEKEE***<br><br>*Wacha wazi kwa matumizi ya ofisi ya IRB.*	***Matumizi ya Ofisi ya IRB Pekee:***<br>Tarehe ya Idhinisho:<br>Tafsiri ya idhini ya IRB iliyoidhinishwa :<br>Jina la PI:<br>Nambari ya IRB.

Namba ya swali	Maswali	Majibu	Kodi	Nenda
		____________________<br>Sijui/Siwezi kusema	#	

Peana hati za idhini kwenye ***Microsoft Word PEKEE***<br><br>*Wacha wazi kwa matumizi ya ofisi ya IRB.*	***Matumizi ya Ofisi ya IRB Pekee:***<br>Tarehe ya Idhinisho:<br>Tafsiri ya idhini ya IRB iliyoidhinishwa :<br>Jina la PI:<br>Nambari ya IRB.

Sehemu 3: WANAYOYAPITIA KWENYE KUTOA HUDUMA NA MITAZAMO YA WANAUMME WALIOTAHIRIWA

Namba ya swali	Swali	Majibu	Kodi	Nenda
301	Umetahiriwa?	Ndio<br>La<br>Sijui/siwezi kusema	1<br>2<br>97	302<br>301a<br>Seh. 4
301.a	*[Iwapo hajatahiriwa*] kwani hujatahiriwa?	Mila<br>Hakuna mtu ameniambia kuihusu<br>Maamuzi yangu mwenyewe<br>Daktari alinishauri kwa sababu ya kiafya<br>Nyingine (taja husika)----------	**1**<br>2<br>3<br>4<br>**7**	Yote:nenda Seh. 4
302	Kwa ninni uliamua kutahiriwa?<br><br>(Chagua yote yahusikayo)	Mila<br>Niliambiwa na mtu<br>Chaguo langu mwenyewe – Kupunguza maambukizi ya HIV<br>Chaguo langu mwenyewe – Kupunguza maambukizi ya STIs<br>Chaguo langu mwenyewe – kuboresha tendo la kujamiana<br>Daktari alinishauri kwa sababu ya kiafya<br>Nyingine (taja husika)----------<br>Sijui/siwezi kusema	1<br>2<br>3<br>4<br>5<br>6<br>#<br>97	

Peana hati za idhini kwenye Microsoft Word PEKEE<br><br>Wacha wazi kwa matumizi ya ofisi ya IRB.	**Matumizi ya Ofisi ya IRB Pekee:**<br>Tarehe ya Idhinisho:<br>Tafsiri ya idhini ya IRB iliyoidhinishwa :<br>Jina la PI:<br>Nambari ya IRB.

Namba ya swali	Swali	Majibu	Kodi	Nenda
303	Ulikuwa na umri upi wakati wa kutahiriwa?	Mtu mzima<br>Mtoto mchanga<br>Mtoto mdogo mwenye umri wa miaka 1-4<br>Umri wa kubaleghe 5-14<br>Aliyebaleghe wa miaka 15-17<br>Sijui/siwezi kusema	1<br>2<br>3<br>4<br>5<br>97	303a<br>304<br>304<br>304<br>303.a<br>304
303.a	Ulitahiriwa kitambo kipi?	Wiki moja iliyopita<br>Mwezi mmoja uliopita<br>Miezi sita iliyopita<br>Mwaka mmoja uliopita<br>Iwapo zaidi ya mwaka mmoja uliopita, andika mwaka …<br>**Sijui/siwezi kusema**	1<br>2<br>3<br>4<br>5<br>97	
304	Ulitahiriwa wapi?	Jina la kituo/eneo:___________<br>nyumbani<br>Sijui/siwezi kusema	1<br>2<br>97	 307
305	Ulitahiriwa na mhudumu wa Afya au daktari wa kienyeji/mhudumu asiye wa kiafya?	Mhudumu wa Afya<br>Daktari wa kienyeji<br>sijui/siwezi kusema	1<br>2<br>97	306a-c<br>307<br>307
306a	Mhudumu wa Afya alikufahamisha kuhusu athari na faida za kutahiriwa kabla ya kukubali kufanyiwa utaratibu huo?	ndio<br>la<br>sijui/siwezi kusema	1<br>2<br>97	
306b	Mhudumu wa Afya alikupa maelezo yoyote yahusianayo na kipindi	ndio	1	

Namba ya swali	Swali	Majibu	Kodi	Nenda
	chote cha kupona yahusianayo na muda wa kukaa bila kujamiana, iwapo unahitaji kurudi kwa maangalizi, n.k?	la<br>sijui/siwezi kusema	2<br>97	
306c	Alikuambia ufanye/usifanye nini?<br>[*chagua yote yanayohusika*]	Usijamiane<br>Rudi kwa maangalizi<br>Maangalizi ya kidonda<br>Hatari ya HIV hata kama umetahiriwa<br>Kipindi cha kupona<br>Hakuna maelezo yalipeanwa<br>Sijui/ sikumbuki<br>Nyingine (taja husika): _____________	1<br>2<br>3<br>4<br>5<br>6<br>97<br>#	
307	Ni maelezo yepi yanaafiki majadiliano yako na wanaumme wengine kuhusu kutahiri?<br>Jibu moja tu	Sizungumzi na wanaumme wengine kuhusu kutahiri<br>Wakati wanaumme wengine wanaanzisha mada hiyo, ninazungumza nao kuhusu wanaumme kutahiriwa, lakini na marafiki wangu au watu wa familia yangu tu.<br>Wakati wanaumme wengine wanaanzisha mada hiyo, ninazungumza nao kuhusu wanaumme kutahiriwa, haijalishi ni marafiki wangu au watu wa familia yangu. Hata watu ambao siwafahamu vizuri<br>Naanzisha mada hiyo na wanaumme wengi ili kuwatia moyo wapate kutahiriwa, lakini na wanafamilia yangu au marafiki zangu<br>Naanzisha mada hiyo na wanaumme wengi ili kuwatia moyo wapate kutahiriwa, hata kama ni watu	1<br>2<br>3<br>4<br>5	309<br>308<br>308<br>308<br>308

Namba ya swali	Swali	Majibu	Kodi	Nenda
		siwafahamu vizuri.		
308	ULIZA IWAPO SWALI TANGULIZI NI = 2-5<br><br>Ni nini wanaumme ambao hawajatahiri hukuuliza kuhusiana na kutairiwa?<br><br>SOMA MAJIBU HAYA YOTE<br><br>KUNA UWEZEKANO WA MAJIBU MENGI	Mahali ambapo ulifanyiwa<br><br>Kuhusu kipindi cha mashauriano kabla ifanyike<br><br>Kuhusu uchungu wowote ulihisi wakati wa utaratibu huo<br><br>Kuhusu maelezo uliyopewa baada ya utaratibu huo<br><br>Kuhusu uchungu wowote uliohisi kipindi cha kupona<br><br>Kuhusu athari ya wakati wa utaratibu wa kupona kwenye shughuli zako za kila siku mfano kufanya kazi au kuenda shuleni<br><br>Kuhusu athari ya utaratibu wa kupona kwenye maisha yako ya kujamiana, iwapo una mwenza<br><br>Mwenza wako alisema nini baada ya kuona umme wako kwa mara ya kwanza baada ya kutahiriwa?<br><br>Ulipitia hali yoyote ya kutia aibu baada ya kutahiriwa?<br><br>Ulihisi mwenye furaha baada ya kutahiriwa?	1<br>2<br>3<br>4<br>5<br>6<br>7<br>8<br>9<br>10	
309	Ulipatwa na matatizo yoyote siku baada ya kufanyiwa utaratibu huo?	ndio<br>la<br>Sijui/Hawezi kusema	1<br>2<br>97	309a<br>310<br>310

Namba ya swali	Swali	Majibu	Kodi	Nenda
309a	[Iwapo alipata tatizo sana katika 404]<br><br>Ulikumbwa na tatizo gani?<br><br>[KUBALI MAJIBU ZAIDI YA MOJA]	Uchungu kwenye fumbatio<br>Kuvimba sana<br>Hematoma<br>Kuvuja damu<br>Maambukizi<br>Tatizo kukojoa<br>Kuchelewa kwa kidonda kupona<br>Tatizo na mwonekano<br>Ajali kwa glansi<br>Uumme kutosimama<br>Nyingine (taja husika) ________________	1<br>2<br>3<br>4<br>5<br>6<br>7<br>8<br>9<br>10<br># 	
309b	Tatizo lililotajwa hapo juu lilitatuliwa vipi?	Nilipokea matibabu katika kituo cha Afya ambamo MC ilifanyika.<br>Nilipokea matibabu katika kituo kingine<br>AE ilijitatua bila matibabu<br>Nilijitibu AE<br>Nilipokea matibabu kutoka kwa mwanafamasia (Hakuona daktari)<br>Nilipokea matibabu kutoka kwa daktari wa kienyeji<br>Tatizo halijatatuliwa<br>Sijui/siwezi kusema	1<br>2<br>3<br>4<br>5<br>6<br>7<br>97	
309c	Tatizo Ilikuchukua muda upi kuitatua?	Siku 1-3<br>Siku 4-6<br>Zaidi ya siku 7	1<br>2<br>3	

*Peana hati za idhini kwenye **Microsoft Word PEKEE***<br><br>*Wacha wazi kwa matumizi ya ofisi ya IRB.*	***Matumizi ya Ofisi ya IRB Pekee:***<br>Tarehe ya Idhinisho:<br>Tafsiri ya idhini ya IRB iliyoidhinishwa :<br>Jina la PI:<br>Nambari ya IRB.

Namba ya swali	Swali	Majibu	Kodi	Nenda
		Ni janga kubwa. Bado halijatatuliwa<br>sijui/siwezi kusema	4<br>97	
310	Ni nini unachukulia kama faida muhimu kwako, iwapo kuna yoyote, tangu kutahiriwa?<br>[Chorea mpaka majibu 3]<br>[Usisome orodha, chagua majibu yaliyopeanwa]	hakuna [ichoree peke yake]<br>Kwa kijumla ni bora [ichoree peke<br>Kujizuia kutoka kwa maambukizi ya HIV<br>Kujizuia kutoka kwa maambukizi ya magonjwa mengine ya Zinaa<br>Usafi bora<br>Ongezeko la kuridhika kwa tendo la ndoa<br>Imelinda mila<br>Imelinda Imani ya kidini<br>sijui/siwezi kusema [ichoree peke yake]	1<br>2<br>3<br>4<br>5<br>6<br>7<br>8<br>97	
311	Unaweza kupendekeza kutahiri kwa wanaumme kwa wanafamilia au marafiki zako?	ndio<br>la<br>sijui/siwezi kusema	1<br>2<br>97	
312	Umepeana rufaa kwa mtu yeyote kwa ajili ya kutahiri?	ndio<br>la<br>sijui/siwezi kusema	1<br>2<br>97	
313	Unaweza kupendekeza kutahiri kwa mwanafamilia au rafiki?	ndio<br>la<br>sijui/siwezi kusema	1<br>2<br>97	

*Peana hati za idhini kwenye **Microsoft Word PEKEE***<br><br>*Wacha wazi kwa matumizi ya ofisi ya IRB.*	***Matumizi ya Ofisi ya IRB Pekee:***<br>Tarehe ya Idhinisho:<br>Tafsiri ya idhini ya IRB iliyoidhinishwa :<br>Jina la PI:<br>Nambari ya IRB.

Sehemu 4: KUTHIBITISHA HADHI YA KUTAHIRIWA

RA: Pata idhini iliyotiwa sahihi kutoka kwa wateja tarajiwa ili kuthibitisha hadhi yao ya kutahiriwa.

401	Hadhi ya kutahiriwa baada ya kuangalia	Ametahiriwa vizuri<br>Ametahiriwa nisu<br>Hajatairiwa	1<br>2<br>3	Sitisha<br>402
402	**Lini, iwapo utawahi, pata kutahiriwa ? (Chagua moja)**<br>**SOMA SKELI**<br>**CHAGUA JIBU MOJA TU**	Kamwe<br>Ndani ya wiki 2 zijazo<br>Ndani ya wiki 2 - 4<br>Ndani ya miezi 1 - 3<br>Ndani ya miezi 4 - 6<br>Ndani ya miezi 7 - 12<br>Baada ya miezi 12 kutoka sasa	0<br>1<br>2<br>3<br>4<br>5<br>6	

Sitisha mahojiano. Mshukuru mtu huyo kwa kushirikiana. Chorea kiwango cha kushiriki hapa chini. Iwapo kuna majibu unahisi hayawezi kutegemewa, andika kwenye maswali na sabubu inayofanya wewe kusema kuwa hayawezi kutegemewa kwenye ‘’maoni’’.

500) Kiwango cha kushiriki

Peana hati za idhini kwenye ***Microsoft Word PEKEE*** <br>*Wacha wazi kwa matumizi ya ofisi ya IRB.*	***Matumizi ya Ofisi ya IRB Pekee:*** Tarehe ya Idhinisho: Tafsiri ya idhini ya IRB iliyoidhinishwa : Jina la PI: Nambari ya IRB.

____ 1. juu ______ 2. kati ____ 3. kidogo

Maoni kuhusu jambo/swali lolote

