# Supplementary material for: Prevalence of male circumcision in four culturally non-circumcising counties in western Kenya after 10 years of program implementation from 2008 to 2019
Source: PLoS One. 2021 Jul 15;16(7):e0254140. doi: 10.1371/journal.pone.0254140 (PMC8281999; doi:10.1371/journal.pone.0254140)
Supplement: S3 File — (PDF) [file pone.0254140.s003.pdf]

*Submit Consent Documents in **Microsoft Word ONLY***<br><br>*Leave blank for IRB Office Use.*	***IRB Office Use Only:***<br>Approval Date:<br>Approved Consent IRB Version No.:<br>PI Name:<br>IRB No.

Study Title: A Population-Based Survey to Validate Male Circumcision Coverage in Four Counties in Kenya
Principal Investigator: Dr. Anthony Gichangi
IRB No.:
PI Version Date: Version 1; April 15, 2019

INFORMED PARENTAL CONSENT FORM

Study Questionnaire

Cluster number________________________________

Household number____

Quality Control Checklist

QC1) Interview Status (Circle only one):

Fully Completed - 1

Partially Completed - 2

Refusal or Partially Completed due to Refusal - 3

Total number of visits: _________

QC2) Language of Interview (Circle only one):

Dholuo 1
Kiswahili 2
English 3
Other 97

QC3) Research Assistant Self Check (field), print first name: ______________________

Date: ___________________________

QC4) Field Supervisor Check (field), print surname: ___________________________

Date: ___________________________

QC5) Field Supervisor Sit-in (field), print surname: ___________________________

QC6) Field Supervisor HH Back-check (field), print surname: ___________________________

QC7) Field Manager Check (field), print surname: ___________________________

Date: ___________________________

QC8) Field Manager HH Back-check (field), print surname: ___________________________

QC9) # of missing values found & fixed by Field Supervisor: _____________________

Supervisory Comments: ____

Questionnaire Entry Completed: ___________________________

Date: ___________________________

Okan'g 1: PENJO MAG ANYUOLA KOD NGIMA JACHIWRE

Namba	Penjo	duoko	Codes	Go To
101	In gi higni maromo nade<br>(higni)?<br>Chein'g nyuol	__________igni<br>_________________		
102	Ratiro mari mar nyombo?	Pod ok anyombo , aonge chotna (misumba)<br>Pod ok anyombo; adak kod chotna<br>Pod ok anyombo, antiere kod chotna, ok adak kode<br>Asenyombo, adak kod chiega<br>Asenyombo, ok adak kod chiega<br>An kod chola<br>Wapogore kod chiega<br>Ok an'geyo<br>Otamore duoko	1<br>2<br>3<br>4<br>5<br>6<br>7<br>97<br>98	
103	In ja dhok mane	JA-EMBU=1<br>JA-KALENJIN=2<br>JA-KAMBA=3<br>JA-KIKUYU=4<br>JA-KISII=5<br>JA-MUA=6<br>JA-LUO=7<br>JA-MASAI=8<br>JA-MERU=9<br>JA-MIJIKENDA=10<br>JA-SOMALI=11<br>JA-TAITA TAVETA=12		

Namba	Penjo	duoko	Codes	Go To
		SWAHILI=13<br>MAMOKO=96<br>________________<br>(LER MATIN)<br>…………………………………		
104	Isedak kae maromo nade<br>[nying anyuola/town/gwen'g/aluora]?<br>[NDIKI KATA ILUOR DUOKO]	HIGNI ADI ________ kata<br>higa< 1<br>ok an'geyo/ok anyal wacho	 1<br>97	
105	Ilemo e din mane?	Christianity<br>Muslim<br>Judaism<br>Dinde machon mag piny<br>Onge maleme<br>mamoko (ndiki): ______________	1<br>2<br>3<br>4<br>5	
106	Tiegruok mamalo mar somo mari?	Ok asomo<br>Ja-lowa primary (klas 1-4)<br>Ja-upper primary (klas 5-7)<br>junior secondary (klas 8-10)<br>senior secondary (klas 11-12)<br>moloyo klas 12	1<br>2<br>3<br>4<br>5<br>6	107<br>106a<br>106a<br>65a<br>106a<br>106a
106a	[kanene idhi e skul nyaka106] En tiegruok/okan'g mane mamalo mane ichopie?	______________		
107	Bende iseko tiyo tich moro amora e dweche apar gi ariyo mokalo ma ochuli pesa kata gimoro amora ma ok pesa?	Eee<br>ooyo	1<br>2	

Namba	Penjo	duoko	Codes	Go To
		ok an'geyo<br>otamore	97<br>98	
108	Bende isetiyo tich moro amora e odichienge abirio ma okalo ma ochuli pesa kata gimoro amora ma ok en pesa?	Ee<br>ooyo<br>ok an'geyo<br>otamore	1<br>2<br>97<br>98	
109	Inyalo wacho ni tich ma itiyo sani ni chalonade?<br>[*luor achiel kende*]	Tich ma ondika<br>Tich ma andikorae kenda<br>Amanyo tich<br>Aonge kod tich<br>An nyathi sikul (seche te)<br>An nyathi sikul(seche moko)<br>Mamoko, ndiki_________	1<br>2<br>3<br>4<br>5<br>6<br>#	
110	**Isetiyo tich moro amora e dweche apar gi ariyo ma okal ma omiyo idhi e county moro ma opogore kod county ma idakie?**	Ee<br>ooyo<br>ok an'geyo<br>otamore	1<br>2<br>97<br>98	
111	**Isetiyo tich moro amora e odichienge abirio ma okalo ma omiyo idhi e county moro ma opogore kod county ma idakie?**	Ee<br>ooyo<br>ok an'geyo<br>otamore	1<br>2<br>97<br>98	
112	**Ka sani pod imanyo tich, e dwech achiel ma okalo, manyo tich oseteri e county ma ok en county ma idakie?**	Eee<br>Ooyo<br>Ok amany tich<br>ok an'geyo<br>otamore	1<br>2<br>3<br>97<br>98	Dhi e 113
113	**[ka Eee] nying county/counties?**	…………………………………………	#	

*Submit Consent Documents in **Microsoft Word ONLY***<br><br>*Leave blank for IRB Office Use.*	***IRB Office Use Only:***<br>Approval Date:<br>Approved Consent IRB Version No.:<br>PI Name:<br>IRB No.

Namba	Penjo	duoko	Codes	Go To
		………………..County		

Okan'g 2: TERO NYANGU: N'GEYO WECHE KOD PACH CHACHIWRE

Namba	Penjo	Duoko	Codes	Go To
201	Bende ne isewinjo wach tero nyangu ka pok abiro kae?' [RA: ka jachiwre oduoko ni ooyo e dhok mar nonro to ipenje gi dho ngere]	yes<br>ooyo [ndikni itiyo kod dho ngere]	1<br>2	202a<br>Sec 3
202	[ka Eee 201] iwinjo ni en ango? [luor duoko momiyi]	Golo pien moumo dho duon'g ngato<br>Gen'go ne dichuo tuo mar ayaki<br>Rem man'gech seche ma in'gade<br>Rem man'geny ka osetiek n'gade<br>Oweyo bedo malungore<br>Omedo bedo malungore<br>Duon'g moter nyangu ler gande<br>Ok an'geyo/ok anyal wacho	1<br>2<br>3<br>4<br>5<br>6<br>7<br>97	
203	Bende in'geyo yawuoni mamoko ma nyithindu kata weteni moseter e nyangu?	Eee<br>Ooyo<br>Ok an'geyo/ok anyal wacho	1<br>2<br>97	204a<br>Sec 3<br>Sec 3
204a	[*ka ee*] adi?	Ndiki kar kwangi: ___________		204b
204b	[ka ee kar 203] ka en moloyo achiel, ka ikawo ranyisi gi achiel motenore kodi, nene otere nyangu e kar thieth kata kod jalony mar chiwo thieth?	Kar od thieth<br>Jan'geto ma jadala<br>Jatend kanisa<br>Jaduon'g mar dala<br>mamoko (ndiki) _________________<br>ok an'geyo/ok anyal wacho	1<br>2<br>3<br>4<br>#<br>97	

Okan'g 3: NGIMA MAPILE PILE KOD HUMA MA JOK MA OTER NYANGU NI GODO

Namba	Penjo	Duoko	Codes	Go To
301	Bende oseteri nyangu?	Eee<br>Ooyo<br>Ok an'geyo/ok anyal wacho	1<br>2<br>97	302<br>301a<br>Sec. 4
301.a	*[ka pok otere nyagu*] Ango ma omiyo pok itero nyangu?	Chike mag piny<br>Onge n'gat ma osenyisa wachni<br>En yiero mara<br>Laktar nene okwera kaluwore kod ngimana<br>mamaoko (ndiki)----------	**1**<br>2<br>3<br>4<br>**7**	All: go Sec. 4
302	An'go mane omiyo iyie tero nyangu?<br>(yier kaluwore)	Chike mag piny<br>N'gato emane onyisa<br>Nene achiwora awuon- konyo gen'go ayaki<br>Nene achiwora awuon- konyo gen'go touché mag nyach<br>Nene achiwora awuon- konyo medo weche mag riwruok<br>Laktar emane onyisa kaluwore kod ngima mara<br>mamaoko (ndiki)---------<br>ok an'geyo/ok anyal wacho	1<br>2<br>3<br>4<br>5<br>6<br>#<br>97	
303	Ne ija higni adi kane oteri nyangu?	N'gat maduong<br>Nyathi madhoth	1<br>2	303a<br>304

Namba	Penjo	Duoko	Codes	Go To
		Nyathi madirom higni 1-4<br>Mihiya maromo higni 5-14<br>Mihia maromo higni 15-17<br>Ok an'geyo/ok anyal wacho	3<br>4<br>5<br>97	304<br>304<br>303.a<br>304
303.a	Ne oteri nyangu ekinde mane mosekalo?	Juma mokalo<br>Dwe achiel mokalo<br>Dweche auchiel mokalo<br>Higa achiel mokalo<br>Ka deponi bor moloyo higa 1 to indik higa …..<br>Ok an'geyo/ok anyal wacho	1<br>2<br>3<br>4<br>5<br>97	
304	Nene itero nyangu kanye?	Nying kanyo:___________<br>Ei dala<br>Ok an'geyo/ok anyal wacho	1<br>2<br>97	307
305	Nene oteri nyangu kod jatich migao mar thieth koso kod ajuoga majadala ma ok jatich migao mar thieth?	jatich migao mar thieth<br>ajuoga majadala<br>Ok an'geyo/ok anyal wacho	1<br>2<br>97	306a-c<br>307<br>307
306a	Bende jatich migao mar thieth nenen oleroni ber kata rach mar tero nyangu ka pok ne iyie mondo on'gadi?	Eee<br>Ooyo<br>Ok an'geyo/ok anyal wacho	1<br>2<br>97	
306b	Bende jatich migao ma rthieth nene olalorekidi eikinde mane ichango motenore gi ndalo mowinjore ikaw kapod ok itimo timbe mag hera kata kane dwarore mondo orang kaka ichango?	Eee<br>Ooyo<br>Ok an'geyo/ok anyal wacho	1<br>2<br>97	
306c	Nene onyisi ni itim an'go koso ni kik itim an'go?	Chungo timbe mag hera(n'gotho)	1	

Namba	Penjo	Duoko	Codes	Go To
	[*yier ma owachni*]	Rango chang mari<br>Rito adholoa<br>Bedo ni pod inyalo yudo ayaki kata koseteri nyangu<br>Ndalo mag chango<br>Onge gima ne okona<br>Ok an'geyo/ok anyal paro<br>mamoko (ndiki): _____________	2<br>3<br>4<br>5<br>6<br>97<br>#	
307	Mane kuom wechegi miyo wa ranyisi kind mbaka misebedogo gi yawuoni mamoko ewach mar tero nyangu?<br><br>DUOKO ACHIEL KENDE	Ok awuyie tero nyangu gi yawuoyi mamoko<br><br>Ka yawuoni mamoko okelo mbaka mar nyangu, alalore kodgi to man aka gin osiepe kata wede maga<br><br>Ka yawuoni mamoko okelo mbaka mar nyangu to alalore kodgi kata ka ok gin wede kod asiepena to bende kata ka gin jok ma akia<br><br>Achako mbaka mar tero nyangu mondo ajiwgo jomoko mondo otime, to mana aka gin wede kod osiepe<br><br>Achako mbaka mar tero nyangu mondo ajiwgo jomoko mondo otime, kata ka ok gin wede kod osiepena	1<br>2<br>3<br>4<br>5	309<br>308<br>308<br>308<br>308
308	PENJI KALUWORE KOD = 2-5<br><br>En an'go ma jok mapok oter nyangu penjiga kaluwore kod tero nyangu mari?<br><br>SOM WECHEGI TE<br><br>DWOKO NYALO BEDO MAN'GENY<br><br>LWOR DUOKOGI	Kuma nene oterae nyangu<br><br>Lalaruok mane ayodo kapok ne oterae nyangu<br><br>Lit manene awinjo eseche mane itera nyangu<br><br>Weche manene osiemago ban'g kane osetere nyangu<br><br>Kind rem mane awinjo eseche manene	1<br>2<br>3<br>4<br>5	

Namba	Penjo	Duoko	Codes	Go To
		adhi mbele kod chango		
		Kakak ngimana nene olokore eyorena mag dak, kaka tich kod skul e kinde mane pod ok atieko chango.	6	
		Kit kaka ne bedo ni pok achango nene omako wach mar mar riwruok kod nyako ma chotna	7	
		Kaka nene ochalone ochot mara chien'g manene oneno nyanguna	8	
		Gima nene omyiya wichkuot ban'ge kanenene osetera nyangu	9	
		Gima nene omiya migosi bang kanene osetera nyangu	10	
309	Bende nene ibedie kod lit kata chandruok moro amaora ban'g kane oseteri nyangu	Eee<br>Ooyo<br>Ok an'geyo/ok anyal wacho	1<br>2<br>97	309a<br>310<br>310
309a	[ka chandruok matutu to dhi e 404]<br><br>En kit chandruok kata thagruok mane maniyuod?<br><br>[MI THUOLO MAR DUOKO MAN'GENY]	Rem man'geny	1	
		Thulo kata kuot mar duon'gna ahinya	2	
		Remo makudore e duon'gna	3	
		Chwero remo man'geny	4	
		adhola	5	
		winjo mapek seche mane alayo	6	
		adhola nokawa ndalo man'geny chango	7	
		duon'gna nenen opogore kod kaka nene ochalga	8	
		adholo nomako wi duon'gna	9	
		nene ok anyal chiek (mar chund	10	

Namba	Penjo	Duoko	Codes	Go To
		machun'g)<br>mamoko (ndiki) _______________	#	
309b	Ere kaka chandruok manene oyudi malo kae ni nene okony?	Nayudo thieth e kar thieth manene oterae nyangu<br>Nayudo thieth kar thieth mamoro<br>Kama ne lit nochango maonge thieth<br>Nathiedho kenda kuma nelitno<br>Nene ayudo thieth e od yath (ne ok orange kod laktar)<br>Nene ayudo thieth kuom ajuoga mar piny<br>Pod ok ayudo konyruok<br>On an'geyo/ok anyal wacho	1<br>2<br>3<br>4<br>5<br>6<br>7<br>97	
309c	Nene okawo ndalo marom nade mondo ikonyri?	Ndalo 1-3<br>Ndalo 4-6<br>Moloyo ndalo 7<br>Pod ok ayudo konyruok<br>On an'geyo/ok anyal wacho	1<br>2<br>3<br>4<br>97	
310	En an'go maduong minyalo wacho ni tero nyagu okonyigodo? [oyiene luoro duoko nyaka adek(3)]<br>[KIK ISOMNE, YIER DWOKO	Onge [luor mae kende]<br>Ober mana moromo [luor mae kende]<br>Aritora ne yudo kute mag ayaki	1<br>2<br>3	

Namba	Penjo	Duoko	Codes	Go To
	MOWACHNI]	Aritora ne yudo kute mag nyach<br>Omedona ler<br>Mit mar nindo kod nyako omedore<br>Otimore kaka chike mag dhowa drwaro<br>Otimore kaka chike mag lembwa kod din dwaro<br>Ok an'geyo/ok anyal waco [luor mae kende]	4<br>5<br>6<br>7<br>8<br>97	
311	Bende inyalo nyiso osiepeni kata wedeni mondo oyie otergi nyangu?	Eee<br>Ooyo<br>Ok an'geyo/ok anyal wacho	1<br>2<br>97	
312	Bende isegande nyiso ngato mondo adhi otere nyangu?	Eee<br>Ooyo<br>Ok an'geyo/ok anyal wacho	1<br>2<br>97	
313	En an'go manyalo moni tamori nyiso watni kata osiepni mondo odhi otere nyangu?	Eee<br>Ooyo<br>Ok an'geyo/ok anyal wacho	1<br>2<br>97	

Okan'g 4: NONRO MAR RANGO ADIERA MAR TERO NYANGU

RA: Bedgi otas mar lwedo ma chachiwre omiyigodo thuolo mar none

401	Chal mar nyangu mare kaseneno kendo amulo duon'gne	Otere nyangu tee<br>Otere nyangu nus<br>Pok otere nyangu	1<br>2<br>3	Giko<br>402
402	**Kar an'go ma digombie mondo oteri nyangu? (yier achiel)**<br>**SOM RANYISI**<br>**YIER MANA DUOKO ACHIEL**	Ok adwar<br>Wige 2 mabiro<br>Ekind wige 2 kod 4 mabiro<br>Dwe 1 nyaka 3 mabiro<br>Dweche 4 nyaka 6 mabiro<br>Dweche 7 nyaka 12 mabiro<br>Bang dweche 12 kochakore sani	0<br>1<br>2<br>3<br>4<br>5<br>6	

Giko mar nonro. Gone jachiwre erokamano. Lour okan'g mar chiwruok mare piny ka.kadebed ni nitiere dwoko ma iparo ni ok adiera ndiki kar weche misambla ka inyiso ni en penjo mane kendo gi gimomiyo iparo ni gionge ratiro.

500) okang mar chiwruok

*Submit Consent Documents in **Microsoft Word ONLY*** <br>*Leave blank for IRB Office Use.*	***IRB Office Use Only:*** Approval Date: Approved Consent IRB Version No.: PI Name: IRB No.

____ 1. maber _____ 2. Man ediere ____ 3.matin

Weche moko kata penjo mag masambla

