# Supplementary material for: Prevalence of male circumcision in four culturally non-circumcising counties in western Kenya after 10 years of program implementation from 2008 to 2019
Source: PLoS One. 2021 Jul 15;16(7):e0254140. doi: 10.1371/journal.pone.0254140 (PMC8281999; doi:10.1371/journal.pone.0254140)
Supplement: S1 Table — (PDF) [file pone.0254140.s004.pdf]

Variable name	Format	Variable label	Variable value labels
Unique_id	string	Unique participant id	
eacode	numeric	KNBS enumeration area code	
residence	numeric	Participant residence	1 Rural
			2 Urban
agecat	numeric	Age of participant in years - category	1 10-14 Years
			2 15-29 Years
finalweights	numeric	Final survey weights	
maritalstatus	numeric	Marital status of the participant	1 Never Married
			2 Married
			3 Separated/Divorced/Widowed
			4 No response
education	numeric	Level of education of participant	1 Primary & below
			3 Secondary
			4 Post-Secondary
religion	numeric	Religion of the participant	1 Christian
			3 Other
employment	numeric	Level of employment of participant	1 Employed
			2 Unemloyed
ethnic	numeric	Ethnic group of participant	1 Luo
			2 Non-Luo
age_cat1	numeric	Age of participant in years - category	1 10-14 Years
			2 15-19 Years
			3 20-24 Years
			4 25-29 Years
verifiedmcstatus	numeric	Verified MC status?	0 No
			1 Yes
verifiedmc	numeric	Verified MC status of the participant	1 Circumcised
			2 Partially circumcised
			3 Circumcised
			4 Declined
county	numeric	County of residence	1 Homa Bay
			2 Kisumu
			3 Migori
			4 Siaya
reportedmc	numeric	Reported participant male circumcision status	1 Circumcised
			2 Uncircumcised
verifiedmc_final	numeric	Verified MC status of the participant	1 Circumcised
			2 Uncircumcised
age	numeric	Age of participant in years	29-Oct
medicalcirc	numeric	Type of circumcision	1 Medical
			2 Non-Medical
circumcisedby	numeric	If circumcised, who circumcised you?	1 Health worker
			2 Non-health workers
