# Supplementary material for: Prevalence of male circumcision in four culturally non-circumcising counties in western Kenya after 10 years of program implementation from 2008 to 2019
Source: PLoS One. 2021 Jul 15;16(7):e0254140. doi: 10.1371/journal.pone.0254140 (PMC8281999; doi:10.1371/journal.pone.0254140)
Supplement: Attachment — Submitted filename: Response to Reviewers.pdf [file pone.0254140.s007.pdf]

Point-by-point Response to Review Comments [POE-D-20-37607R1] - [EMID:39a51a8e4b4f1cb1]

Manuscript Title: Prevalence of male circumcision in four culturally non-circumcising counties in western Kenya after 10 years of program implementation from 2008 to 2019

<table>
<tr><th>No.</th><th>Comments</th><th>Response</th></tr>
<tr><td colspan="3">Response to comments by Reviewer No 1</td></tr>
<tr><td>1</td><td>In the re-written manuscript, the DMPPT estimates are stated in the text as % over-estimation relative to the survey for each of the Counties in the study. The DMPPT estimates themselves are not listed anywhere. For ease of reference, the VMMC results table should include a column with the DMPPT estimate and can then have a column with the % over-estimation relative to the survey. Otherwise, it is too difficult for a reader to pull these figures out of the text without having a table to refer to.</td><td>We have included a table that compares the 2019 DMPPT 2 modelled MC prevalence estimates with the population survey results for the same year (Table 3 on page 16; Line 256-258).</td></tr>
<tr><td colspan="3">Response to comments by Reviewer No 2</td></tr>
<tr><td>2</td><td>This is a revision of an analysis investigating the prevalence of male circumcision and associated characteristics in four counties in Kenya. This manuscript has been previously reviewed and it appears in the response to reviewers that the authors have addressed the concerns with the changes reflected in the edited manuscript. I recommend reviewing the tables and suppressing cells less than 5 to protect privacy.</td><td>Tables 2 and 3 have marital status variable which has category “Divorced, separated or Widowed” which has cell values less than 5. Please note that this category cannot be merged with either of the two remaining categories, that is, Never married and Married because they are standalone. Therefore, this category (Divorced, separated or Widowed) of marital status with cell values less than 5 can only remain as is and only used in descriptive and weighting cases only.</td></tr>
</table>

Dr. Elijah Odoyo-June, MBchB, Msc, PhD.
For Male Circumcision prevalence survey team, Kenya
