# Supplementary material for: Prevalence of male circumcision in four culturally non-circumcising counties in western Kenya after 10 years of program implementation from 2008 to 2019
Source: PLoS One. 2021 Jul 15;16(7):e0254140. doi: 10.1371/journal.pone.0254140 (PMC8281999; doi:10.1371/journal.pone.0254140)
Supplement: Attachment — Submitted filename: Response to Reviewers.docx [file pone.0254140.s006.docx]

**Point-by-point Response to Review Comments [PONE-D-20-37607]**

**Manuscript title:** Prevalence of male circumcision in four culturally non-circumcising counties in western Kenya after 10 years of program implementation from 2008 to 2019

**Point by point response to reviewers**

| **No.** | **Comments** | **Response** |
| --- | --- | --- |
| **Journal Requirements and editor’s comments** | | |
| 1 | Carefully consider the comments about the interplay between the observed data and the modelling estimates in your reporting. It is not clear whether this plays a large or a small role in the work and the conclusions. | We have stated in the results that the DMPPT2-Model overestimated the MC prevalence. The DMPPT2 modelled MC prevalence in 2016 were 5-10% higher than the observed prevalence results in 2019 despite 3 years of uninterrupted VMMC services in the intervening period. |
| 2 | Reduce commentary and speculation about partial circumcision as this was almost wholly unobserved. | Commentary on partial circumcision has been reduced in the discussion |
| 3 | Please include additional information regarding the survey or questionnaire used in the study and ensure that you have provided sufficient details that others could replicate the analyses. For instance, if you developed a questionnaire as part of this study and it is not under a copyright more restrictive than CC-BY, please include a copy, in both the original language and English, as Supporting Information, or include a citation if it has been published previously. | Copies of the questionnaire in English, Kiswahili and Dholuo have been included as supporting information. |
| 4. | Please include further description of the questionnaire development process in your methods section. | Additional information on questionnaire development is now included in the methods section. |
| 5. | In the Methods, please discuss whether and how the questionnaire was validated and/or pre-tested. If these did not occur, please provide the rationale for not doing so. | Information on pre-testing of questionnaire has been added under methods. |
| 6. | In statistical methods, please refer to any post-hoc corrections to correct for multiple comparisons during your statistical analyses. If these were not performed please justify the reasons. Please refer to our statistical reporting guidelines for assistance (<https://journals.plos.org/plosone/s/submission-guidelines.#loc-statistical-reporting>). | The survey weights were calculated per county and not for all the four counties in this analysis. Thus, multiple comparison analysis was not necessary. The manuscript has been updated to reflect this. |
| 7. | Please include your amended statements within your cover letter; we will change the online submission form on your behalf. | Amended funding statement is included in the cover letter. It reads as follows: This survey was funded by the President’s Emergency Plan for AIDS Relief (PEPFAR) through the U.S. Centers for Disease Control and Prevention (CDC) under the terms of Cooperative Agreement # GH001469. The funders had no role in study design, data collection and analysis, decision to publish, or preparation of the manuscript. |
| 8. | We note you have included a table to which you do not refer in the text of your manuscript. Please ensure that you refer to Table 5 in your text; if accepted, production will need this reference to link the reader to the Table. | Table 5 has been referenced in the manuscript |
| **Additional Editor Comments** | | |
|  | 1) 1. There are several places where the text is not clear about the role and predictions of the DMPPT2 modelling estimates. In the back ground and introduction much greater clarity about the mathematical modelling issues is needed to better understand the context for the work. • Second sentence of abstract introduction needs revision – this reviewer could not understand • No context for the “lower population-based MC prevalence” is given in the abstract – I think the context of overly optimistic model projections is needed • Introduction: More precision in the language is needed to make it clear when estimates are “model estimates” and when estimates are from observed data. For example mention of “VMMC program saturation” – it is not clear whether this is estimated saturation or field (observed) saturation. | Relevant areas of the manuscript including abstract, introduction, discussion and conclusion have been edited to improve clarity. |
|  | Another area that needs clarity is whether “MC” combines both traditional and VMMC. It appears the MC reported is almost all VMMC, but this needs to be discussed. | This has been clarified in the manuscript.  We have stated in the methods sections that reported circumcisions were coded as medical if conducted by a health worker otherwise they were considered non-medical. The contribution of non-medical circumcision to the total circumcisions reported low for the 10-14-year age band (ranging from 2.1 in Siaya to 6.9% in Migori) but high for the 15-29 year age band (ranging from 3.1 in homa bay to 28.4 in Migori) |
|  | Methods: “none selection” should be “non-selection” | Thanks for correction. ‘none selection” has been changed to “non-selection’ |
|  | Statistical: The sampling design and the sample size were very similar in different regions. It seems surprising that the 95%CI for 10-14 yo in Migoro was ~ +/- 15%, but for Siaya was +/- 8%. Typically, the SE would not change so dramatically for prevalence in the range ~55% to ~75%. | The desired sample size for each county was not achieved. Therefore, accrued sample for each county was re-weighted to reflect the required sample size. The re-weighting increased the SE more markedly for counties with a bigger gap between the accrued and desired sample size. |
|  | Tables: • In Table 1 and Table 2 row% should be replaced by col%, as there is little interest in comparison between regions. • In Table 1: Please change to % interviewed within each subgroup; similarly the last two rows could be entered as % fof the two rows above rather than as separate rows. | Thanks for the correction. Tables 1 and Table 2 have been revised as suggested. We have changed Row% to Col%. |
|  | Discussion: • Several instances of problems with references and words that were left in from editing “(insert coverage…)” • Line 274-278: This is the first-time details are provided about the modelled estimates. It would be helpful to include this in the introduction. The statement that the observed estimates were lower than the modelled estimates is incorrect – because they are within the range provided for the 10-14 yos, simply on the lower end. The range is, of course very wide (so wide as to be not very useful?). • Line 287ff. This reviewer understood that traditional MC in the South African is typically partial, and there were extremely few partial circumcisions reported. It would be helpful in the commentary in the discussion about the potential role of traditional circumcision in Migori if this could be clarified, as it seems somewhat in conflict with the explanation offered. | Thanks for correction; Bookmark Errors! and editorial comments have been excluded from the discussion text.  DMPPT2 modelled estimates have been included in the introductions and referenced in the discussion.  Yes, partial circumcisions were very few; 0.2% (9/3410) of the respondents who underwent genital examination. The observed cases were distributed randomly across the counties regardless of the prevalence of non-medical circumcision and with no clustering among non-medically circumcised respondents compared to those circumcised medically. Circumcises among culturally circumcising residents of Migori county are not typically partial. |
| **Response to comments by Reviewer No 1** | | |
| 1 | **Title** Comparing the title to the abstract, it was difficult to tell whether the purpose of the study was to ascertain VMMC prevalence in four counties of Kenya (title) or to validate the DMPPT2 (abstract). It is fine for there to be a secondary purpose but best to be consistent between title and abstract as to the main purpose of the study. | The purpose of the survey was to ascertain the MC prevalence in the four target counties and secondarily use the observed MC prevalence to validate the DMPPT2 modeled estimates.  We edited the introduction to reflect this. |
| 2 | **Abstract**  The abstract is inconsistent as described above. Introduction implies that the purpose is to validate DMPPT2, but Results only discuss VMMC prevalence in the four counties and Conclusion does not help readers interpret the results in terms of whether DMPPT2 was validated.  The abstract should be revised to ensure it makes sense as a stand-alone document. I recommend reading it with fresh eyes and revising for clarity. Here are some issues that stood out to me as a new reader:  "80% saturation" -- what does this mean? Why is 80% a point of saturation?  "Estimates from the DMPPT2 included unlikely MC coverage approaching 100% without commensurate decline in VMMC uptake." -- I could not understand the meaning of this sentence  From reading conclusions I could not tell whether the survey results did or did not agree with the DMPPT2 estimates. Clearly state this and discuss possible reasons why or why not. | We have edited the abstract for better alignment with the title and clarified that the DMPPT 2 model overestimated the prevalence of MC especially for the 15-29-year age band. |
|  | **Introduction**  "The program achieved 92% of its target" -- please clarify what the target is. | This sentence has been edited as follows to improve clarity.  Kenya’s VMMC program achieved 92% of its service delivery target for the first 5-year national strategic plan (2008–2013) and met its annual targets for the second strategic plan (2014–2019) thereby reporting over two million cumulative circumcisions as of September 2018. |
|  | **Methods** line 109 "using probability proportional to size sampling" is unclear. Is this probability in proportion to the total population, males age 10+, land area? Please specify and, if possible, provide a reference to the complete method.  line 111 "All households in the sampled EAs were listed to help update NASSEP IV" is unclear. Did a staff member manually enumerate each HH? Was this list used directly or merged with the NASSEP list? If merged, how was it deduplicated?  The survey weights description was excellent and very clear except for this part on Lines 173-174: "Finally, the weights were adjusted to ensure consistency with the projected population figures." It was not clear to me what this meant methodologically.  The long sentence starting on Line 176 is missing a subject, and could generally be re-written for readability/clarity: County population MC prevalence (both verified and self-reported) was calculated by multiplying the value of each participant’s survey response by the corresponding nonresponse-adjusted [***MISSING SUBJECT***] summing up the products across all units (clusters and strata) and finally dividing by the sum of all weights. | Line 109 updated to show that the cluster population of males 10-29 years was used in PPS sampling. Reference provided.  Line 111 updated – a KNBS staff and an RA visited selected EAs and manually enumerated HHs, the new HH listing formed the new sampling frame and no need for deduplication.  Line 173-174 has been revised to read “The survey final weights were normalized so that the total final weights equals to the total sample size”  This sentence has been revised to read - County population MC prevalence (both verified and self-reported) was calculated. Multiplying the value of each participant’s survey response by the corresponding nonresponse-adjusted weights, then summing up the products across all units (cluster) and finally dividing by the sum of all weights per county. |
|  | **Results** Given the claim that the survey resolved discrepancies with DMPPT, Results should also include results from DMPPT and whether they agree with the data given sampling uncertainties in the survey. Some of the DMPPT results are included in Discussion instead which makes for an awkward flow.  Table 3 is the "meat" of the paper and it is very large and difficult to navigate. A figure and a map would be welcome. Maybe a map of counties colored by prevalence, overlayed with dots representing EAs also colored by prevalence. | The DMPPT2 modelled mc prevalence for the end of 2016 have been included and cited as ref no 7.  The DMMPT modelled MC prevalence estimates were as at the end of 2016 (three years before the before the population survey). For men 15-29 years, the modelled estimates were higher than observed prevalence despite 3 years of sustained VMMC services and high uptake by this age band before the survey. Sustained High VMMC uptake among age bands that had attained high MC prevalence approaching 100% by DMPPT2, coupled with lower observed population MC prevalence 3 years later – point to DMPPT2 overestimation of prevalence thereby resolving the discrepancy.  Table 3  KNBS maintains the shapefiles for EAs and they don’t share that data and thus impossible to map. We have included a bar graph (Fig 1) showing MC prevalence by county and the two age bands. |
|  | Discussion  As best I understand, PEPFAR no longer recommends circumcision for ages 10-14 due to higher risk of adverse events. The paper should address this. For example, the observation that 15-19yo boys are 2x more likely to become circumcised than 10-14yo boys should be discussed in the context of the recent guidance.  The Discussion section has multiple instances of "(insert coverage for these counties)" and "Error! Bookmark not defined."  DMPPT results should be in Results instead of Discussion.  A large section of the Discussion focuses on incomplete circumcision. This part of the paper is both confusing and concerning. It speaks of nine individuals with short but not entirely removed foreskins, says four of them stated they were uncircumcised, and for some reason deduces that four of them were partially circumcised. Why four and not five?  ls in this study was extremely small, so much so that there may not be significant implications for surveys from this finding. It seems important to point that out. To address this formally, the authors could perform sensitivity analyses to determine (1) if the partially circumcised participants would have any meaningful impact on the survey results, and (2) if partial circumcision would meaningfully reduce the coverage of VMMC. If it is very rare and not a driver of survey bias or coverage, then it does not seem appropriate to single it out as a main topic of Discussion. | WHO and PEPFAR guidelines de-emphasizing VMMC for ages 10-14 years due to higher risk of adverse events and consenting challenges have been cited and included in the discussion (Ref # 18 and 19).  Thanks for correction; Bookmark Errors! and editorial comments have been excluded from the discussion text.  DMPPT results have been removed from the discussion and are now cited in the introduction and Results section.  Write up on partial circumcision has been edited and reduced to limit speculative content |
|  | | |
